# Corruption, poverty and inflation as enablers of AMR in low- and middle-income countries: finding the link and addressing the gaps

**DOI:** 10.1093/jacamr/dlaf221

**Published:** 2025-11-28

**Authors:** Kenneth Chukwuebuka Egwu, Maryam Abdulkarim, Shadrach Chinecherem Eze, Nduka Precious Agenu, Abdulmajeed Opeyemi Agboola, Oluchi Mbamalu

**Affiliations:** Department of Pharmacy, Karshi General Hospital, Federal Capital Territory, Nigeria; The Community BRIDGE Health Initiative, Awka, Anambra State, Nigeria; Department of Clinical Infection Microbiology and Immunology, University of Liverpool, Liverpool, UK; Department of Pharmaceutical and Medicinal Chemistry, College of Pharmacy, Afe Babalola University, Ado-Ekiti Ekiti State, Nigeria; Faculty of Pharmaceutical Sciences, Ghent University, Ghent, Belgium; Department of Obstetrics and Gynaecology, Federal Medical Centre, Asaba, Delta State, Nigeria; Department of Public Health, Kwara State University, Kwara State, Nigeria; The Community BRIDGE Health Initiative, Awka, Anambra State, Nigeria; Department of Global Health, Faculty of Medicine and Health Sciences, Stellenbosch University, Cape Town, South Africa; Global Surgery Division, Faculty of Health Sciences, University of Cape Town, Cape Town, South Africa; IVAN Research Institute, University of Nigeria, Enugu, Enugu State, Nigeria

## Abstract

Antimicrobial resistance (AMR) is a global health challenge that disproportionately affects countries and regions with limited resources. Current efforts to address AMR largely emphasize antimicrobial stewardship (AMS), community engagement and scientific research, while often overlooking systemic factors such as corruption, poverty and inflation, which significantly influence the capacity of individuals and communities to respond effectively. In this article, we suggest that AMR responses must extend beyond traditional AMS and community engagement to adopt a genuinely people-centred approach—one that empowers individuals not only to achieve optimal health but also to navigate broader social and economic constraints. To our knowledge, this is the first report to critically examine the combined impact of corruption, poverty and inflation on AMR.

The discovery of penicillin about a century ago revolutionized healthcare, with antibiotics now central to infectious disease treatment. In recent times, however, AMR has become a major threat to this breakthrough, unevenly threatening global health security. In 2021, AMR was responsible for nearly 5 million deaths, well on the way to a projected 10 million deaths annually by 2050.^1,2^ A recent update shows that AMR could lead to three deaths every minute between 2025 and 2050,^2^ underscoring the need to address this burden. The disproportionate distribution of AMR and the high mortality recorded in low- and middle-income countries (LMICs) go beyond limited awareness and encompasses various systemic factors across political and economic contexts that negatively influence purchasing power and standard of living, impacting the infection-prevention and health-seeking behaviours of the population.^3^

Although efforts have focused on addressing AMR through multifaceted AMS programmes, infection prevention and control (IPC) initiatives, risk communication and community engagement, the influence of systemic factors such as governance, corruption, poverty and inflation, as well as culture and context, as enablers of AMR are often overlooked.^3–6^ Studies conducted across high-income countries and LMICs highlight that poor governance and corruption strongly correlate with observed AMR rates, compared with antibiotic use volumes.^5–8^ Along with poor governance and poverty in LMICs is a lack of access to basic amenities such as water, sanitation and hygiene (WASH), healthcare, nutritious food, habitable environments and clothing.

Globally, the percentage of people living in extreme poverty increased from 8.9% in 2019 to 9.7% in 2020, largely in low-income countries and driven by the COVID-19 pandemic.^9^ Poverty exposes individuals to environments conducive to the spread of infectious disease. In Nigeria, limited resources associated with poverty encourages underdosing of antimicrobials as individuals ration resources to meet the cost of medicines.^10^ Studies in Tanzania, Kenya and Uganda support this, with participants taking only partial antibiotic courses due to financial constraints.^11^

Poverty is linked to socio-economic status, a key determinant of access to WASH—not only in sub-Saharan Africa, in which access to WASH services is among the lowest, but also in other LMICs.^12,13^ This link results in limited WASH coverage in underprivileged areas, where the burden of WASH-related diseases is significantly growing.^12,13^ Poverty also decreases optimal care for infection treatment, leaving individuals with lower purchasing power, limited options for their healthcare needs in uncertain and unprotected economic conditions, and a pressing need for a quick fix to avoid taking time off work and hence maintain income-earning capacity. Among the public in many LMICs, antibiotics are misunderstood as offering this ‘quick fix’ to address unavoidable structural shortcomings,^14^ further encouraging AMR development and spread. Studies in LMICs and high-income countries, while acknowledging the impact of antibiotic usage on AMR, have opined that AMR is not only determined by poverty and use levels.^5–7^

Among the many under-researched factors contributing to AMR spread are governance and corruption. Studies across low-, middle- and high-income countries highlight the influence of these two factors on antibiotic use and resistance.^15–18^ Of the $7.5 trillion allocated for healthcare globally, $500 billion—more than sufficient to achieve universal health coverage by 2030—is lost to corruption.^18^ Using data sourced in Europe, in 2015 Collignon *et al.*^5^ reported corruption as a key determinant of antibiotic resistance. In the Philippines, corruption has been found to cause delay in newborn vaccination, lower immunization coverage, and deter people from using public health facilities. In Bangladesh, corruption in the form of bribery takes place during physician recruitment, thereby affecting their distribution and compromising healthcare access.^19^ In Africa, corruption poses a monumental challenge to the healthcare system; billions of dollars meant for public healthcare are lost annually through mismanagement,^18^ diverting vital resources away from the public. The losses are staggering—enough to fund the national healthcare budgets of countries like Nigeria, Ghana and Kenya for 6 to 10 years. This leaves healthcare facilities floundering, and millions of citizens with limited or no access to healthcare. Corruption limits resources for antimicrobial regulation and stewardship, exposing society to the dangers of inappropriate use.^5^

In addition, inflation, related to governance and political decisions, limits healthcare access. Research has documented the effect of inflation and the resultant increase in commodity prices on health outcomes.^20,21^ Uncontrolled surges in medication prices and healthcare costs result in patients seeking less expensive alternatives from non-professional drug vendors, exposing them to harm and danger. Inflation in Burundi, Malawi, Angola and Nigeria currently ranges from 22% to almost 40%,^22^ with far-reaching consequences for AMR prevention: increasing the cost of AMR stewardship and IPC implementation, and crippling surveillance and community engagement efforts.

The link between AMR, corruption, poverty and inflation is often underestimated, leading to interventions that fail to address these underlying drivers. Collignon *et al.*^5^ already established that corporate governance and corruption drive AMR more than antimicrobial use. In sum, systemic issues of governance and the economy fundamentally affect how communities are empowered to engage meaningfully in AMR responses. When healthcare funds are directed under poor governance, community-level efforts to combat AMR will remain constrained, regardless of how much awareness or education is provided. This is because corruption and poverty create conditions that facilitate antimicrobial misuse. For individuals and businesses that rely on antimicrobial sales for income, particularly in unregulated markets, economic survival often takes precedence over responsible use. This is compounded by regulatory gaps that allow for unchecked antibiotic distribution.

Achieving sustainable progress in tackling AMR requires transparent allocation and use of funds, supported by strong institutional frameworks that prevent corruption. These include strengthened regulatory oversight, economic protection for vulnerable groups, addressing corruption in pharmaceutical supply chains to reduce incentives for inappropriate antimicrobial marketing and distribution, and investment in job creation and economic opportunities to support local production and promote financial empowerment among the population. Ultimately, the fight against AMR must be people-centred, rooted in empowering communities with not just information to combat AMR but also resources for healthy lives and required access to basic amenities and services for infection prevention and control.
